# Activity Regulation by Fibrinogen and Fibrin of Streptokinase from Streptococcus Pyogenes

**DOI:** 10.1371/journal.pone.0170936

**Published:** 2017-01-26

**Authors:** Sian Huish, Craig Thelwell, Colin Longstaff

**Affiliations:** 1 Component development laboratory, NHS Blood and Transplant, Cambridge Donor Centre, Cambridge, United Kingdom; 2 Biotherapeutics Section, National Institute for Biological Standard and Control, South Mimms, Herts, United Kingdom; University of North Dakota School of Medicine and Health Sciences, UNITED STATES

## Abstract

Streptokinase is a virulence factor of streptococci and acts as a plasminogen activator to generate the serine protease plasmin which promotes bacterial metastasis. Streptokinase isolated from group C streptococci has been used therapeutically as a thrombolytic agent for many years and its mechanism of action has been extensively studied. However, group A streptococci are associated with invasive and potentially fatal infections, but less detail is available on the mechanism of action of streptokinase from these bacteria. We have expressed recombinant streptokinase from a group C strain to investigate the therapeutic molecule (here termed rSK-H46A) and a molecule isolated from a cluster 2a strain from group A (rSK-M1GAS) which is known to produce the fibrinogen binding, M1 protein, and is associated with life-threatening disease. Detailed enzyme kinetic models have been prepared which show how fibrinogen-streptokinase-plasminogen complexes regulate plasmin generation, and also the effect of fibrin interactions. As is the case with rSK-H46A our data with rSK-M1GAS support a “trigger and bullet” mechanism requiring the initial formation of SK•plasminogen complexes which are replaced by more active SK•plasmin as plasmin becomes available. This model includes the important fibrinogen interactions that stimulate plasmin generation. In a fibrin matrix rSK-M1GAS has a 24 fold higher specific activity than the fibrin-specific thrombolytic agent, tissue plasminogen activator, and 15 fold higher specific activity than rSK-H46A. However, in vivo fibrin specificity would be undermined by fibrinogen stimulation. Given the observed importance of M1 surface receptors or released M1 protein to virulence of cluster 2a strain streptococci, studies on streptokinase activity regulation by fibrin and fibrinogen may provide additional routes to addressing bacterial invasion and infectious diseases.

## Introduction

Streptokinase (SK) is a bacterial plasminogen (Pgn) binding and activating protein widely used as a therapeutic thrombolytic agent [1]. The commercial molecule is isolated from Group C streptococci and is able to activate Pgn in solution and is not fibrin specific [2]. Subsequent development of Pgn activator drugs, so-called second and third generation thrombolytics highlighted the desirability of fibrin specificity and led to the development of the tissue Pgn activator (tPA) family of drugs [3]. However, other microbial Pgn activators have a preference for activating fibrinogen (Fgn) or fibrin-bound Pgn which is associated with pathogenesis [4, 5]. Thus studying the mechanisms of Pgn activation by microbial activators is helpful in better understanding infection [6] and has been applied to the development of fibrin specific thrombolytics, such as staphylokinase [7]and engineered versions of streptokinase [8, 9].

The fundamental principles of the SK mechanism of Pgn activation are understood and have been explored in some detail, focussing on the Group C therapeutic variant, SK-H46A [10]. It is accepted that SK binds Pgn to form the active SK•Pgn complex, which activates further Pgn molecules to generate the protease plasmin (Pm). The initial SK•Pgn complex requires no cleavage of zymogen Pgn at Arg^561^-Val^562^, to generate an active site in bound Pgn, but rather the N-terminal Ile residue of SK forms a salt bridge with Asp740 of Pgn to induce an active conformation by a “molecular sexuality” mechanism [11, 12]. The active SK•Pgn complex generates Pm (Pm) which results in the formation of the more active SK•Pm complex [13]. Detailed mechanistic studies have labelled this pathway of Pm generation, through sequential formation of SK•Pgn and SK•Pm complexes, the “trigger and bullet” mechanism [14]. *Streptococcus pyogenes* variants, which are Group A streptococci (GAS), are considered to be more virulent and cause more disease than Group C species, including toxic shock syndrome, necrotising fasciitis, throat infections and impetigo [5, 15]. An important aspect of increased virulence involves interactions with the host Pgn activation system [16], dependent on variations in SK molecules, and the presence of Fgn binding proteins (the M family of proteins) and Pgn binding proteins (Pgn-binding group A streptococcal M-like proteins, or PAMs) and other Pgn receptors [17–19]. There is no simple relationship between any particular genotype of streptococci and virulence or type of disease and variations between bacterial strains are known [4]. For example GAS cluster 2a has M1 Fgn binding proteins but no PAM, which is present in cluster 2b [20]. The interaction of M1 proteins with host Fgn which then binds Pgn is proposed as mechanism of localising Pm activity to the cell surface or evading host immune responses [15, 20], but details of how the interactions of SK•Pgn or SK•Pm with Fgn or fibrin might regulate Pm generation are not fully understood. We have studied activation of recombinant SK from group 2a (rSK-M1GAS) as a SK variant from a strain that uses M1 to bind Fgn during Pgn activation. The kinetics of rSK-M1GAS have been studied with Fgn and fibrin and compared with rSK-H46A, a recombinant version of the therapeutic, non-fibrin-specific Group C-derived SK variant, and with tPA. The results of these studies will provide a better understanding of how Fgn and fibrin might influence the regulation of Pgn activation and evolution of virulence amongst streptococci strains. Comparison of rSK-M1GAS and tPA will also provide insights into the design of fibrin-specific thrombolytics.

## Materials and Methods

### Protein expression and purification

Recombinant SK (rSK) rSK-H46a was cloned and expressed as previously described [21]. The coding region for mature rSK from the M1 type GAS (rSK-M1GAS) was amplified by PCR using genomic DNA from Streptococcus pyogenes strain SF370 (ATCC 700294) as template, and cloned and expressed in the same way as rSK-H46a. N-terminal deletion mutants of rSK-H46a and rSK-M1GAS, missing the coding region for amino acids 1–59, were created by PCR and cloned and expressed the same way as full length rSK. All rSK variants were expressed as N-terminal SUMOstar fusion proteins (SUMOstar Expression Kit from LifeSensors, PA), from transformed T7 Express *lysY E*. *coli*. SUMOstar fusion proteins, which include an N-terminal His-tag sequence, were purified from culture lysates by Ni-affinity chromatography using a 1 ml HisTrap HP column equilibrated with 20 mM Tris-HCl, 0.5 M NaCL, 40 mM imidazole, pH 7.7 at room temp on an AKTA Purifier (GE Healthcare, Amersham, UK). The SUMOstar fusion tag was cleaved using SUMOstar protease according to the manufacturer’s protocol. SUMOstar protease cleaves precisely at the junction between the SUMOstar tag and the N-terminal amino acid of the target protein. This ensures the integrity of the N-terminal Isoleucine of full length SK, crucial for the “molecular sexuality” mechanism of action. Protein concentrations were determined by amino-acid analysis or using Coomassie Plus reagent relative to a standard curve of full-length SK quantified by amino acid analysis (peformed by Alta-Bioscience, Birmingham, UK).

### rSK activity assays

Amidolytic activity of Pm (the WHO 3^rd^ International Standard for Pm. 97/536, NIBSC, South Mimms, UK) alone or in complex with rSK H46a, rSK M1GAS, rSK H46a del 1–59 and rSK M1GAS del 1–59 was determined in solution against the chromogenic substrate for Pm, S-2251 (, H-D-Val-L-Leu-L-Lys-pNA (Chromogenix, Milan, Italy) at 37°C using a plate reader (Molecular Devices, Stamford, CA) to calculate initial hydrolysis rates before 10% substrate depletion. K_M_ and k_cat_ values were then calculated for each complex by non-linear regression of Michaelis Menten curves using GraphPad Prism (GraphPad Software, San Diego, CA). In all instances, 1.35 nM final concentration Pm or microplasmin was used with a two-fold molar excess of SK. An extinction coefficient of 2500 Abs M^-1^ was determined for pNA under these experimental conditions and used to convert rates from Abs min^-1^ into M s^-1^. Where present, Fgn (Calbiochem, Billerica, MA) was added to the reaction mixture to a final concentration of 3 mg/ml.

rSK activation of Pgn was investigated using either glu-Pgn (Hyphen Biomed, Neuville-sur-Oise, France) or lys-Pgn (a gift from Immuno, Vienna, Austria). Pm generation was followed by hydrolysis of S-2251, as described above and rates of Pgn activation calculated from the slopes of plots of absorbance versus time squared, as previously described [22] (see also automated version app [23]). Potency estimates for rSK, in IU/ml, were calculated from Pgn activation rates relative to the WHO 3^rd^ IS for Streptokinase, 00/464, NIBSC, South Mimms, UK, using a parallel line bioassay analysis. Specific activities (IU/μg) were calculated from potency values using the protein concentrations of the rSK preparations. Where stated in results, reaction mixtures also included ranges of concentrations of Pgn or Fgn. tPA was the WHO 3^rd^ IS, 98/714, NIBSC, South Mimms, UK. Modified Fgn or fibrin degradation products (FDP) were included in some Pgn activation assays as described in results. Oxidised Fgn was prepared using chloramine T according to [24] and mixtures of FDP were prepared from clot lysis reaction mixtures generated by following the European Pharmacopoeia method for tPA activity determination [25] and pooling the mixed fibrin degradation products collected from the reaction mixture after 6, 20, 60 and 300 min of lysis. Two separate pools were made from different time courses, labelled FDP-1 and FDP-2. Cyanogen bromide Fgn degradation products were from Technoclone, Vienna, Austria.

Fibrinolysis assays on purified fibrin clots were investigated in microtitre plates. Fibrin clots were made in blocked microtitre plate wells by adding a solution of 3.0 mg/ml Fgn containing 100 nM Glu- or Lys- Pgn (60 μl) to a 40 μl solution of 20 nM thrombin and rSK (concentration given in Results). Clotting and lysis were monitored at 405 nm and time courses analysed using an R script [26] to calculate time to 50% lysis. Where present, tranexamic acid (Sigma-Aldrich, Poole Dorset, UK) was included in the clotting mixture over the range of final concentrations given in Results.

### Modelling and simulations

A model for rSK Pgn activation in the presence of Fgn was made based on the “trigger and bullet” mechanism proposed by Bock and co-workers (e.g.[14]), as described in Results. Parameters for rate constants and reactant concentrations for this model were entered into the simulation software package Gepasi [27, 28] and time courses simulated to generate data for pNA absorbance generation over time. To match model simulation and data collection methods as closely as possible, rates of Pm generation were calculated (in pM s^-1^) from the simulated data from plots of time squared versus absorbance change of pNA. A custom script written in R [29], was used to calculate rates of Pm and plot figures [30, 31] from data sets of real and simulated kinetic data over ranges of Pgn, Fgn and at 0.4 or 1.6 nM rSK. R scripts and data files are provided in Supporting Information.

## Results

### rSK interactions with Pm

The mechanism of action of SK includes interactions with Pgn, to create an active SK•Pgn complex, and with Pm to generate a SK•Pm complex with altered activity compared with Pm alone [32]. Table 1 summarises a series of experiments focussing on the activity of Pm on the chromogenic substrate S-2251 when free and in stoichiometric complex with rSK-H46A and rSK-M1GAS. Also shown are deletion variants of both SK molecules lacking N-terminal residues 1–59, which prevent N-terminal Ile insertion and activation by the “molecular sexuality” mechanism.

**Table 1:** Pm activity against S-2251, free and in complex with rSK variants.

Pm in complex with	k_cat_ s^-1^ (SEM)	K_M_ μM (SEM)	k_cat_/ K_M_ mM^-1^s^-1^ (SEM)
**Pm alone**	25.5 (± 1.9)	368 (± 10)	69.3 (± 2.0)
**rSK-H46A**	60.4 (± 3.8)	361 (± 10)	170 (± 4.2)
**rSK-M1GAS**	26.7 (± 2.4)	156 (± 60)	171 (± 10.5)
**rSK-H46A del 1–59**	25.1 (± 2.0)	377 (± 20)	66.6 (± 2.4)
**rSK-M1GAS del 1–59**	25.6 (± 1.6)	426 (± 20)	64.4 (± 2.0)
**Pm + Fgn**	14.5 (± 4.2)	1600 (1003)	9.13 (10.0)
**rSK-H46A + Fgn**	70.3 (± 5.7)	400 (± 10)	160 (± 6.0)
**rSK-M1GAS + Fgn**	64.7 (± 12.6)	400 (± 70)	159 (± 16.9)

The results presented in Table 1 show that both rSK-H46A and rSK-M1GAS stimulate Pm activity to the same degree, as the k_cat_/K_M_ for the amidolytic substrate is increased approximately 2.5 fold (from 70 to 170 mM^-1^s^-1^). Interestingly, the mechanism underlying this stimulation is different for the two full length rSK variants as can be seen from the individual k_cat_ and K_M_ values. rSK-H46A stimulates Pm activity by increasing the k_cat_ against S-2251, whereas rSK-M1GAS improves the K_M_ for the substrate. Results with deletion variants lacking residues 1–59 highlight the absolute requirement for the N-terminal region of rSK for both rSK-H46A and rSK-M1GAS, suggesting they both operate by the accepted “molecular sexuality” mechanism. In the presence of a physiological concentration of 3 mg/ml Fgn, the K_M_ of Pm for S-2251 is made worse, as expected since Fgn is a competing substrate. However, there is also a decrease in k_cat_, indicating a more complex type of inhibition. Both rSK-H46A and rSK-M1GAS protect Pm from inhibition, and as the data in Table 1 show, this is achieved by influencing both k_cat_ and K_M_ (compare the bottom 3 rows). Overall, it can be seen that there are differences in the way rSK-H46A and rSK-M1GAS interact with Pm and modulate activity, but a significant effect of rSK binding is to protect Pm from inhibition by Fgn through changes in substrate specificity of bound Pm as noted in other studies on peptide substrates for example [32]. By extension, it is logical to assume that there is also a parallel change in specificity of bound Pm away from fibrin degradation towards activation of Pgn.

### Pgn activation by rSK-H46A, rSK-M1GAS and tPA: stimulation by Fgn

**Fig 1:**
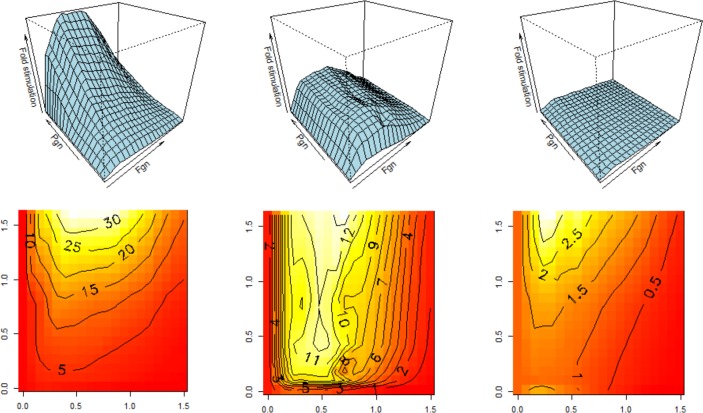
Stimulation of Pgn activation by Fgn by rSK-M1GAS, tPA and rSK-H46A. All panels show the fold-stimulation in rate by Fgn at each Pgn concentration (ratio of rate x[Pgn] at y[Fgn]/rate x[Pgn] with no Fgn) for activators rSK-M1GAS (left), tPA (middle) and rSK-H46A (right). Initial rates of Pm generation were measured by following hydrolysis of S-2251 and determining rates in pM/s Pm formed (z axis), as described in Materials and Methods. The x and y axis shown are log Fgn concentration (0–30.3 μM) and Pgn concentration (0–1.6 μM) respectively. R scripts and associated data are available in Supporting Information.

Based on potency estimates relative to the WHO 3^rd^ IS for Streptokinase (00/464), rSK-M1GAS has approximately 5-fold lower specific activity than rSK-H46a in the absence of any stimulator (12.4 IU/μg compared to 58.2 IU/μg). Previous work has suggested that Fgn may be a weak stimulator of rSK-H46A [33, 34] as for tPA. The effectiveness of Fgn as a stimulator of Pgn activation by rSK-M1GAS, rSK-H46A and tPA was investigated and results are presented in Fig 1, including a range of Fgn and Pgn concentrations, showing level of stimulation by Fgn as surface and contour plots. These results show only very low levels of stimulation by Fgn of rSK-H46A, but more stimulation with tPA and rSK-M1GAS. Both tPA and rSK-M1GAS appear to follow a template mechanism as evidenced by the bell shaped nature of plots of activity stimulation versus log of the Fgn concentration. This kind of relationship strongly suggests the stimulator (Fgn) binds to both activator (tPA or rSK-M1GAS) and Pgn substrate to form a ternary complex. Optimum stimulation occurs at around the physiological concentration of 3–5 mg/ml Fgn for both rSK-M1GAS and tPA, albeit with a different pattern along the Pgn axis. Maximum Stimulation of rSK-M1GAS by Fgn was up to 35-fold at the highest Pgn concentrations used, 1.6 μM, whereas with tPA, stimulation reached a maximum of 12-fold, achieved at lower Pgn concentrations.

**Fig 2:**
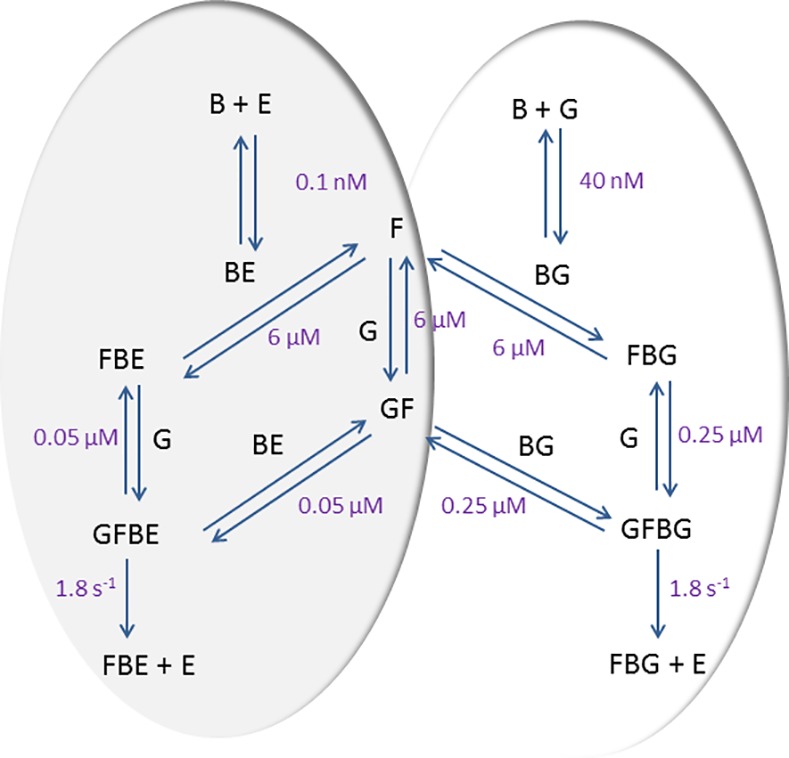
Scheme outlining the pathway of activation of Pgn by rSK-M1GAS and stimulation by Fgn. The scheme follows a trigger and bullet mechanism where an initial activator complex of SK•Pgn (BG) is replaced by SK•Pm (BE) as Pm (E) is generated due to the higher affinity binding of E to SK. Fgn (F) associates weakly with Pgn (G), while formation of active Michaelis complexes, GFBG and GFBE have improved dissociation constants. An improved rate of Pgn activation is achieved by GFBE relative to GFBG due to lower K_M_, while the k_cat_ for formation of Pm is unchanged. Derivation of the constants shown is detailed in Table 2.

Thus, Fgn at normal physiological concentrations would appear to be important regulator of the activity of rSK-M1GAS and mechanistic details were investigated by kinetic model building. Fig 2 shows a scheme developed to explain the activity of rSK-M1GAS with Fgn and Pgn, where the core of the model was based on the work of Bock and colleagues using the “trigger and bullet” mechanism [13, 14, 35]. For simplicity, lys-Pgn was studied, to avoid additional reactions involving rSK-M1GAS binding to different conformers of glu-Pgn, or conversion between glu- and lys-Pgn. In this terminology the trigger is binding of rSK-M1GAS to Pgn to make the initial activator complex (BG in Fig 2 and Table 2), which is replaced as Pm is generated by a more efficient rSK-M1GAS•Pm complex (the bullet, BE) as shown in the shaded area in Fig 2. The published pathway developed for rSK-H46A type enzymes has now been extended to include stimulation by Fgn (F in Fig 2) of rSK-M1GAS. Derivation of the starting parameters is explained in Table 2, which accompanies Fig 2 and optimisation of the fit of model data to kinetic data was accomplished by manual adjustment of model parameters from starting values shown in legend to Table 2. Key features of the model are formation of Fgn-bound active complex of rSK-M1GAS with Pgn as (GFBG) or with Pm (GFBE). The Pm complex is more active by virtue of an improved K_M_ for the substrate, (0.5 μM to 0.06 μM), although the k_cat_ is the same (1.8 s^-1^). This arrangement is mirrored in Table 1 where stimulation of Pm activity by rSK-M1GAS binding is achieved by changes in the active site leading to improvements in K_M_ for S-2251.

**Table 2:** Model Parameters used in the model outlined in Fig 2 and simulated in Fig 3.

Reaction	k_on_^[a]^	k_off_	k_cat_	K_D_ or K_M_	Note
E+S = ES	1e7	3680		368 μM	[b]
ES-> E+P			25.5		[b]
B+G = BG	1e7	0.4		40 nM	[c]
BG+G = BGG	1e7	6		0.6 μM	[d]
BGG-> BG+E			0.1		[d]
G+F = GF	1e7	60		6.0 μM	[e]
F+BG = FBG	1e7	60		6.0 μM	[e]
GF+BG = GFBG	1e7	2.5		0.25 μM	[f]
G+FGB = GFBG	1e7	2.5		0.25 μM	[f]
GFBG->E+FBG			1.8		[g]
E+F = EF	1e7	5		5 μM	[h]
B+E = BE	1e8	0.01		0.1 nM	[i]
F+BE = FBE	1e7	60		6.0 μM	[e]
G+FBE = GFBE	1e7	0.5		50 nM	[j]
GF+BE = GFBE	1e7	0.5		50 nM	[j]
GFBE->E+FBE			1.8		[k]
BE+G = BEG	1e7	3		0.3 μM	[l]
BEG->BE+E	1e7		0.1		[l]

Abbreviations used are: plasmin (E), S-2251 (S), pNA (P), SK (B), Pgn (B), Fgn (F).

[a] association rate constants approximated to 10^7^ M^-1^s^-1^ at 37°C. Values for SK•Pgn complex formation in line k_on_ values of around 10^6^ M^-1^s^-1^ from Biacore measurements at 25°C in [5, 36] and higher estimates for SK•Pm formation in [13].

[b] See Table 1

[c] 40 nM K_D_ is within the range in [5] or 6 nM from [13].

[d] Derived from fitting of Michaelis Menten curves without Fgn, SK•Pgn + Pgn K_M_ = 0.6 μM, k_cat_ = 0.05, This is lower than Boxrud and Bock [14] by ~ 7 fold for rSK-H46A as expected since rSK-M1GAS will be less effective without Fgn (K_M_ was 0.27 μM and k_cat_ 0.31 for Lys-Pgn in [14]). For reaction of SK•Pm we reduced K_M_ by half to 0.3 μM, kept k_cat_ same, so modest increase in activity similar to 1.5 fold improvement in [35].

[e] Upper limits of K_D_ for Fgn Lys-Pgn binding 8.3 μM [37], or an estimate of 0.23 μM from [38].

[f] Starting estimate from Michaelis-Menten fits (as in [c]) at each Fgn concentration. Table 1 suggests Fgn can affect K_M_ and k_cat_, but fits indicate more change to V_max_ (and k_cat_) (see [e]).

[g] The k_cat_ for this reaction accounts for most stimulation by Fgn. To achieve 30 fold stimulation of rate by Fgn k_cat_ increased from 0.1 [d] to 1.8 s^-1^ (additional stimulation from small improvement in K_M_ to 0.25 from 0.6 μM).

[h] Initial estimate based on based on value in [39], but that assumed simple competitive inhibition, which is not the case as seen in Table 1.

[i] This binding of Pm to SK for SK•Pm, tighter than K_D_ for SK•Pgn, similar to biacore data around 0.5 nM for a number of complexes in [5] or 0.3 nM in [13] and is explained in part by increased k_on_ e.g. [13].

**Fig 3:**
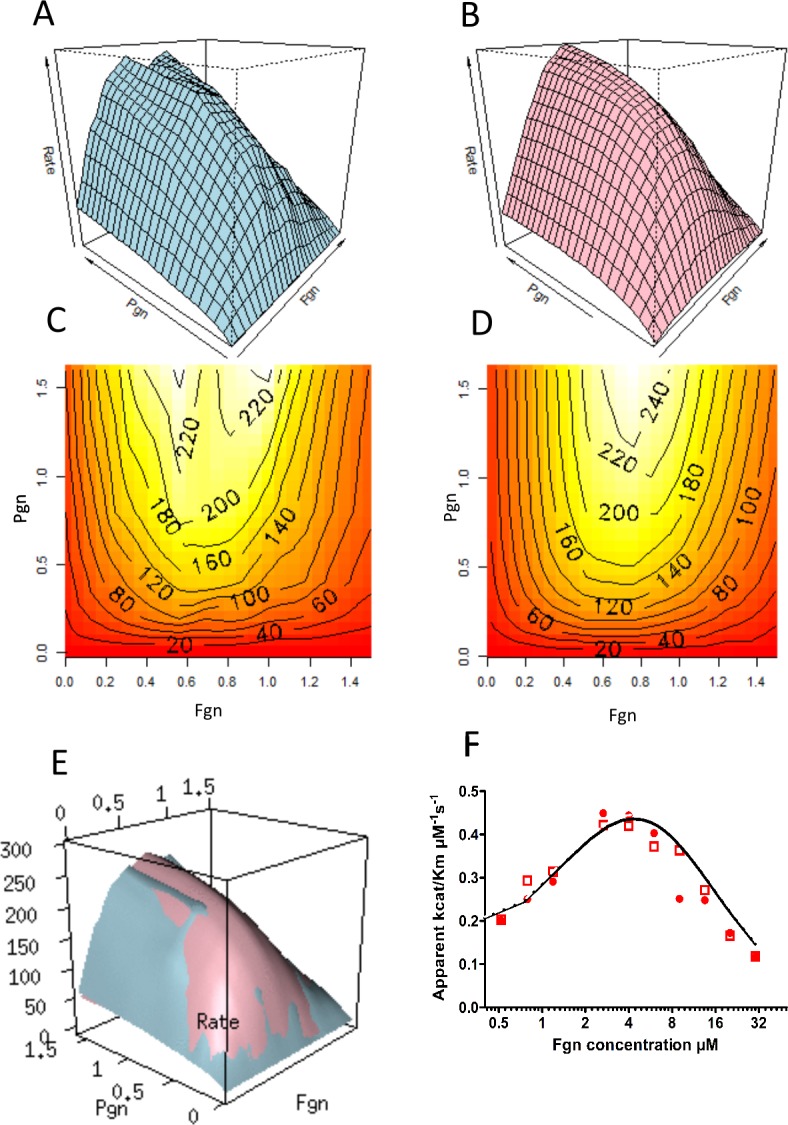
Comparison of Pgn activation data and simulated data for rSK-M1GAS over a range of Pgn and Fgn concentrations. Panels A (experimental data) and C (simulated data) show fitted surface plots of rate of Pm generation plotted against Fgn and Pgn concentrations as shown, for 1.6 nM rSK-M1GAS. Panels B and D present the same results as a surface and contour plots giving rates of Pm production in pM/s against Pgn concentration (0–1.6 μM) and Fgn concentration (log scale for 0–30 μM). Panel E is an overlay of surfaces for the data and simulation shown in panels A and B. Experimental data using rSK-M1GAS at 1.6 (closed circles) and 0.4 nM (open squares) over a range of Pgn concentrations were fitted to the Michaelis-Menten equation to determine k_cat_ and K_M_ values, and calculate k_cat_/ K_M_ at each Fgn concentration and this is shown in panel F. The solid line is for the same values calculated from simulated data using the same ranges of Pgn and Fgn (the lines overlap for 2 hypothetical rSK-M1GAS concentrations of 1.6 and 0.4 nM. R scripts and data files are provided in Supporting Information.

[j] Increase in efficiency of SK•Pm over SK•Pgn due to K_M_ only, in line with Table 1, which is improved from 0.6 μM to 50 nM i.e. 12-fold. This difference important for degree of curvature required to match shapes of curves in Fig 3.

[k] There is no change in k_cat_, 1.8 s^-1^, only change in K_M_ for SK•Pm vs SK•Pgn.

[l] Small improvement without Fgn of SK•Pm vs SK•Pgn activity is due to decrease in K_M_ (from 0.6 to 0.3 μM), in line with Table 1 data.

Fig 3 shows a comparison of real and simulated data as fitted surfaces and contour plots where rates are given in pM s^-1^ Pm generation. These plots differ from those in Fig 1, which show the fold stimulation by Fgn. There is obviously good agreement between model and data in Fig 3A–3E), suggesting the model and constants shown are reasonable explanations for the mechanism of Fgn stimulation of rSK-M1GAS. It is possible that different combinations of parameters could give a good fit but a limited range is allowed to get the correct shape and positions of maxima. Good agreement was also obtained when comparing fits of Michaelis-Menten curves at each Fgn concentration, for rSK-M1GAS at 0.4 and 1.6 nM with fittings to simulated data generated by the model outlined in Fig 2 and Table 2, as summarised by the points and line in the plot of apparent k_cat_ / K_M_ values in Fig 3F.

### Stimulation of rSK-M1GAS by fibrin and fibrin analogues

**Fig 4:**
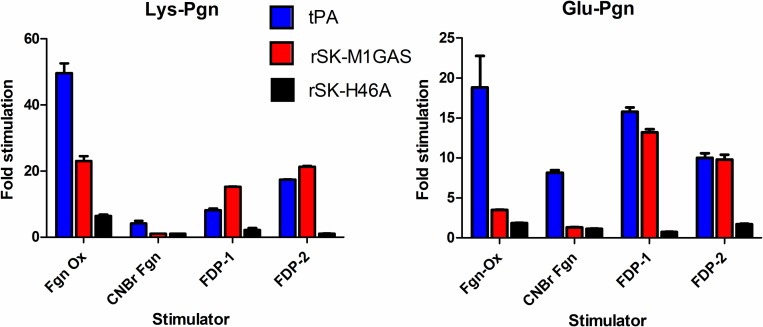
The effect of known stimulators on Pgn activation by tPA, rSK-M1GAS and rSK-H46A. Bars show the degree of stimulation (rate with stimulator/rate without stimulator) for fixed concentrations of Pgn and tPA (blue), rSK-M1GAS (red) and rSK-H46A (black), using both glu- or lys-Pgn as substrate. Abbreviations are Fgn Ox, oxidised Fgn, CNBr, cyanogen bromide fragmented Fgn, and FDP-1 and FDP-2 are pooled samples from separate independent time courses of fibrin degradation products.

Having identified significant levels of stimulation of rSK-M1GAS by Fgn, it was also of interest to investigate stimulation by fibrin and fibrin analogues. Fig 4 summarises data for stimulation of activity of tPA, rSK-H46A and rSK-M1GAS by a number of known stimulators of Pgn activation. Both glu- and lys-Pgn are included as substrates. Generally speaking there is more stimulation of lys-Pgn and there seems to be stimulation of tPA and rSK-M1GAS, but not rSK-H46A, as may be expected from data presented above. In particular, tPA and rSK-M1GAS behave similarly in the presence of mixtures of FDP.

**Fig 5:**
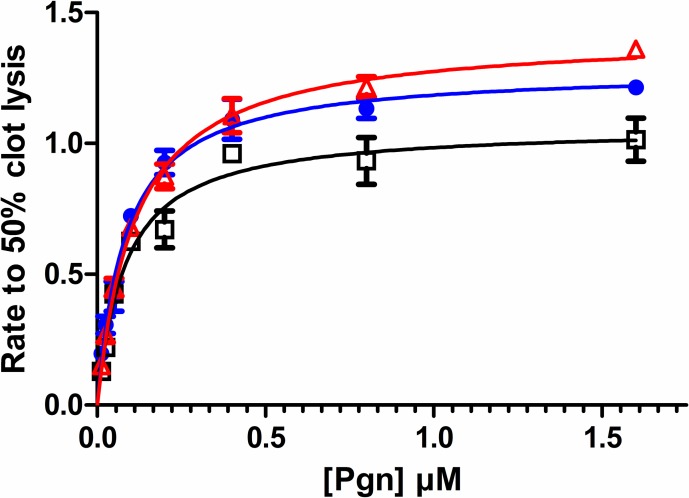
Fibrin clot lysis by tPA, rSK-M1GAS and rSK-H46A over a range of Pgn concentrations. Fibrin clots were prepared using 3 mg/ml Fgn and incorporating Pgn from 0–1.6 μM. Rate of clot lysis was estimated from time to 50% clot lysis, as 1000x 1/time to 50% lysis in seconds. To get similar rates, activator concentrations used were 0.6 M tPA (blue circles), 0.3 nM rSK-H46A (black squares) and 0.02 nM rSK-GASM1 (red triangles). Detailed results from fitting to the Michaelis-Menten equation are presented in Table 3.

The effects of fibrin on the activity of tPA, rSK-H46A and rSK-M1GAS were investigated in fibrin clot lysis assays in a purified system. Clots were formed to contain a range of Pgn concentrations and times to 50% lysis were determined in the presence of a fixed amount of each activator. Fig 5 shows results where concentrations of activators were adjusted to give similar lysis times. Data are expressed here as lysis rate (1000x 1/time to 50% lysis) versus Pgn concentration which allows results to be fitted the Michaelis-Menten equation and provide estimates for apparent K_M_ and V_max_ values, which are summarised in Table 3.

**Table 3:** Kinetic parameters for clot lysis by tPA, rSK-M1GAS and rSK-H46A from data shown in Fig 5.

parameter	tPA	rSK-M1GAS	rSK-H46A
V_max_	1.28	1.42	1.06
K_M_ μM	0.083	0.113	0.082
E_o_ nM	0.6	0.02	0.3
Normalised V_max_/ K_M_ ^a^	25.8	629	43.2
rSK-M1GAS /tPA		24.4	
rSK-M1GAS / rSK-H46A		14.5	

^**a**^Normalised to equivalent molar concentrations for each activator

**Fig 6:**
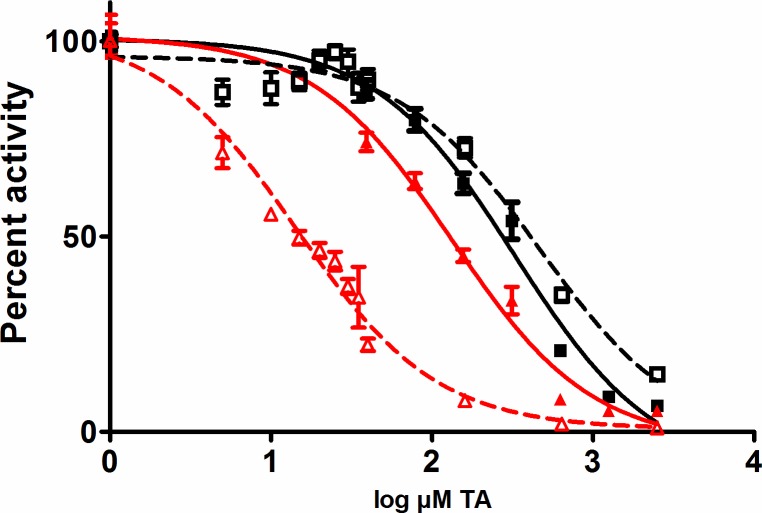
Inhibition of Pgn activation in a Fgn or fibrin environment by tranexamic acid (TA). Data are presented as the % activity remaining relative to activation with no TA where rSK-M1GAS (red symbols) or rSK-H46A (black symbols) is activator. Open symbols and dashed lines are for data in the presence of Fgn and solid symbols and lines are in fibrin. Curve fitting to a 4 parameter model suggests a significant difference between IC_50_for rSK-M1GAS in the presence of Fgn (14.5 μM) and fibrin (133 μM). Inhibition of Pgn activation by rSK-H46A was inhibited at higher TA and there was no significant difference with Fgn or fibrin.

In this assay system the apparent K_M_ was similar for all three activators, 0.08–0.1 μM Pgn, but the concentration of rSK-M1GAS used in the assays was much lower than the other activators, so a normalised V_max_/ K_M_ for rSK-M1GAS was around 15-fold higher than rSK-H46A and 24-fold higher than tPA, in this fibrin-based system. Thus, in the presence of fibrin, rSK-M1GAS on a mole for mole basis, is much more potent than tPA or rSK-H46A. Further aspects of the mechanisms of stimulation of rSK-M1GAS were probed using the lysine analogue TA in systems with Fgn and fibrin. The data shown in Fig 6 indicate a weak binding site involving Fgn and rSK-M1GAS that was disrupted with an IC_50_ of 14.5 (95% confidence interval 11.5–18.3) μM TA. Significantly, the IC_50_ in the presence of fibrin increased around 10-fold to 133 (107–166) μM indicating fibrin binds the components of this system more strongly than Fgn. The high concentrations of TA needed to interfere with rSK-H46A activation kinetics in the presence of Fgn or fibrin were not significantly different, IC_50_ = 465 or 327 μM TA, respectively, and may be due to destabilisation of the SK•Pgn complex or direct inhibition of Pm activity.

## Discussion

S. pyogenes GAS causes a range of infections from mild to life threatening and it is estimated that globally 18 million people harbour serious infections, with disease burden disproportionately affecting poor, disadvantaged or weakened individuals [40]. S. pyogenes binds Fgn via M protein, which is a known virulence factor and is a target for vaccine development [41]. The ability M protein-Fgn complex to provide a template for assembly of SK•Pgn complex generation is a key regulatory feature for Pm generation [20, 42]. By this mechanism, bacterial SK hijacks the host fibrinolytic system to generate Pm which can overcome defensive fibrin barriers produced by the host. A range of bacteria have evolved systems to arm themselves with surface Pm to optimise invasiveness and pathogenicity [43]. Understanding the regulation of Pgn activation by GAS is a possible route to the development of improved antimicrobial therapies, increasingly important as traditional antibiotics become less effective [44]. Other bacterial proteins are also associated with pathogenesis/invasiveness, including Pgn receptors such as alpha-enolase and Pgn-binding group A streptococcal M-like protein (PAM) [19, 36]. In this case, Pgn activation and Pm binding to the cell surface can be achieved without the involvement of Fgn and Fgn binding proteins. PAM has a high affinity for Pgn and it has been suggested that PAM-expressing GAS strains are adapted for environments where Pgn concentrations are low, such as skin, rather than tissues bathed in plasma or fluids rich in Pgn [18]. Naturally, SK is a key virulence marker and SK structure function studies have identified the beta domain of SK as an important factor in accounting for differences between strains [45]. However, pathogenesis is also affected by other host Pgn activators such as urokinase [46], and coagulation proteins affecting thrombin generation also have a role [44].

### Mechanisms of interaction with fibrinogen and fibrin

The focus of the current study was Pgn activation by an SK (rSK-M1GAS) from an S. pyogenes isolate, from cluster 2a, that is known to express M1 surface protein, but not PAM [20]. Thus the rSK-M1GAS is likely optimised for generation of Pm from ternary complexes with Fgn. To better understand this process, we have developed models using kinetic data from rSK-M1GAS and incorporating previous data from non-fibrin-specific group C SK, (rSK-H46A in this work). Observations presented in Table 1 and Fig 1 show evidence of some low level stimulation of rSK-H46A Pgn activation by Fgn, but clearly there is much more stimulation of rSK-M1GAS. Physiological concentrations of Fgn and Pgn would lead to > 30-fold stimulation of rSK-M1GAS activity, which is likely to reflect what would happen during an infection where Pm generated could remain bound to the Fgn on the bacterial cell surface. The models presented in Figs 2 and 3 provide a reasonable explanation for the mechanism of this activation, incorporating stimulation by Fgn and involvement of both SK•Pgn and SK•Pm complexes, as highlighted elsewhere [13, 14]. It was only possible to match data and simulations by involving both SK•Pgn and SK•Pm complexes in the “trigger and bullet” mechanism outlined above. rSK-M1GAS binding and stimulation by fibrin appears to be more significant than Fgn as judged by Figs 5 and 6. rSK-H46A is hardly affected by Fgn or fibrin but rSK-M1GAS interacts more strongly with fibrin than Fgn, as assessed by the concentration TA that is required to displace the complex and block activation (Fig 6). In an environment where fibrin is present it may be expected rSK-M1GAS would preferentially activate Pgn in fibrin, rather than associate with Fgn in solution. However, not addressed in the current work is the stimulation of Pgn activation that may be observed when Fgn is bound to M1 protein. This could be M1 on the bacterial surface or M1 released into solution following cleavage by SpeB a cysteine protease produced by S. pyogenes [47]. Furthermore, it is entirely possible that these two Fgn forms may behave differently as stimulators of rSK M1GAS since free (but not cell surface) M1 can form a network with Fgn, which is a potent activator of the neutrophil αMβ_2_ integrin receptor (Mac1 or CD11b/CD18) [48]. Indeed, release of M1 protein from S. pyogenese cell surface and the presence of M1-Fgn networks is associated with bacterial toxic shock syndrome and necrotising fasciitis [49]. Further investigation on the mechanism of Pgn activation by Fgn bound to cell-associated or free M1 protein would be of interest.

### Structures of activation complexes

Recently published X-ray models of glu-Pgn have clarified the likely structures of complexes with some bacterial Pgn activators. Model building indicates that non-fibrin-dependent molecules like rSK-H46A are able to bind and activate glu-Pgn, whereas other fibrin-dependent activators such as staphylokinase cannot bind the serine protease domain of glu-Pgn because of clashes with kringle 3 [50, 51]. These observations partly explain the fibrin specificity of staphylokinase, which requires a more open, fibrin-bound Pgn structure, rather than conformationally resistant, native glu-Pgn. However, this does not explain why all bacterial activators have not evolved like rSK-H46A to use native glu-Pgn in whatever form presented. There may be some evolutionary advantage to having fibrin or Fgn dependent activity. Problems of conformational resistance to activation do not apply to much of the work presented here which utilised the open, more activatable form of Pgn, lys-Pgn. However, work on glu-Pgn also indicates that rSK-M1GAS is an effective activator of this conformation in the presence of Fgn or fibrin, at slightly lower rates than observed with lys-Pgn (see Fig 4 for example). The nature of the activator complex of SK•Pgn or SK•Pm is not known, and may be similar to the published structure of the microplasmin•SK complex (PDB code 1bml). However, it seems that complexes containing rSK-M1GAS have an available kringle that is used to bind Fgn or fibrin, that can be displaced by TA with an IC_50_ of 14.5 or 133 μM, respectively (Fig 6). The relatively low concentration of TA necessary to block binding to Fgn points to a high affinity TA-binding kringle, probably kringle 1 [52]. As for the Fgn binding partner, previous work on S. pyogenes SK•Pgn complexes propose binding sites on the Fgn D-domain [53]. Stronger interactions with fibrin may reflect additional binding sites uncovered during the change from Fgn to fibrin, possibly with additional kringles in SK•Pgn and SK•Pm taking part in binding. The higher TA concentration required to inhibit activation in fibrin is greater than found in a similar system where tPA was activator with a reported IC50 of 27 μM with lys-Pgn [54]. Thus rSK-M1GAS complexes seem to have a strong affinity for fibrin, consistent with the results presented in Fig 5.

### Fibrin-specific rSK thrombolytics

Previous studies aimed at developing fibrin-specific thrombolytics from engineered SK variants [8, 9], or by identifying novel SK variants [55], have claimed some success. It is clear from the data presented in Figs 4 and 5 that rSK-M1GAS was effectively stimulated by fibrin and fibrin analogues. In comparison to tPA, rSK-M1GAS showed more specificity toward FDP and was less stimulated by modified Fgn (oxidised or CNBr-cleaved) than tPA. Data in Table 3 and Fig 5 shows how potent rSK-M1GAS is in molar terms, with higher specific activity than either tPA or rSK-H46A in the presence of fibrin. Of course, the problem with rSK-M1GAS in any consideration of fibrin specificity is the very effective stimulation in the presence of Fgn (Fig 1). Thus, in vivo, rSK-M1GAS may be expected to have little advantage over rSK-H46A, which has very similar activity in the absence or presence of Fgn or fibrin.

### Streptococcus virulence

Previous kinetic studies on Pgn activation by SK have focussed on the therapeutic molecule from group C strains [14, 56] or cluster 1 or 2b strains S. pyogenes [45, 57], with more limited information available on group 2a strains [5, 58]. The importance of M proteins and the M1 type in particular for S. pyogenes infection has been noted in connection with the unusual virulence and prevalence of the M1T1 strain [59]. Interestingly, this strain carries, in addition to the *emm1*.*0* allele of the M1 gene, several *sda* genes whose streptodornase (a DNase) products facilitate bacterial escape from neutrophil extracellular traps (NETs) composed of DNA and histones mixed with fibrin. Clearly, M proteins, SK and bacterial nucleases play a critical role in regulating bacterial pathogenesis, through interaction with Fgn and fibrin clots and neutrophil integrin receptors [48]. Here we focus on Pm generation and present detailed studies on Pgn activation kinetics and stimulation by Fgn and fibrin of rSK-M1GAS from a cluster 2a strain that expresses M1 surface receptor for Fgn (but no specific Pgn binding proteins). A quantitative understanding of how these components work together with SK variants to generate Pm and promote of invasion and pathogenesis is helpful in identifying new approaches to the treatment of GAS diseases.

## Supporting Information

S1 File Raw data and script files used to prepare Figs 1 and 3 are available for download as a zip file. (ZIP)
